# Choroidal Vortex Vein Drainage System in Central Serous Chorioretinopathy Using Ultra-Widefield Optical Coherence Tomography Angiography

**DOI:** 10.1167/tvst.12.9.17

**Published:** 2023-09-22

**Authors:** Zhonghua Luo, Yue Xu, Kun Xu, Matthew Fan, Ching-Kit Tsui, Xi Lu, Benjuan Wu, Xinyu Zhao, Xiaoyu Tang, Peiqi Wu, Kaixuan Cui, Shanshan Yu, Xiaoling Liang

**Affiliations:** 1 State Key Laboratory of Ophthalmology, Zhongshan Ophthalmic Center, Sun Yat-Sen University, Guangdong Provincial Key Laboratory of Ophthalmology and Visual Science, Guangdong Provincial Clinical Research Center for Ocular Diseases, Guangzhou, China; 2 Yale College, Yale University, New Haven, CT, USA

**Keywords:** choroidal vortex vein drainage system, ultra-widefield optical coherence tomography angiography, central serous chorioretinopathy

## Abstract

**Purpose:**

To evaluate differences in the choroidal vortex vein drainage system (VV) in eyes between patients with central serous chorioretinopathy (CSC) and unaffected individuals using ultra-widefield optical coherence tomography angiography (UWF-OCTA).

**Methods:**

In this cross-sectional observational study, 40 eyes of patients with CSC and 28 eyes of healthy volunteers were included. The analysis involved the use of UWF-OCTA to analyze the proportion of the choroidal vortex vein drainage system (VV%), choroidal thickness, choroidal vascular volume (CVV), and choroidal vascularity index (CVI) of the VV in each drainage quadrant. The location relationship between the leakage points in fluorescein angiography and the VV was also explored.

**Results:**

A within-group analysis of VV% showed a statistically significant difference in the CSC group (*P* < 0.001) but not in the control group (*P* = 0.270). Compared to healthy eyes, CSC eyes had a significantly larger CVV and higher CVI in all regions (all *P* < 0.05). The superotemporal (ST) drainage system had the largest CVV and thickest choroidal layer among the four drainage quadrants (all *P* < 0.05) in CSC eyes. The leakage rate in the ST quadrant was significantly higher than that in the inferotemporal quadrant (*P* < 0.001).

**Conclusions:**

CSC eyes have an asymmetric vortex vein drainage system, with relative hyperperfusion in all VV. Further, the preferential drainage route of the submacular choroid may be the ST drainage system in CSC eyes.

**Translational Relevance:**

Targeting the imbalanced drainage system could be a potential therapeutic approach for CSC.

## Introduction

Central serous chorioretinopathy (CSC) is a common eye condition that primarily affects the macula in young and middle-aged individuals.^1^^,^^2^ The condition is classified as acute CSC (aCSC) and chronic CSC (cCSC) based on the duration of serous retinal detachment (SRD).^3^^,^^4^ The manifestations of aCSC, such as distorted or blurred vision, hypermetropia, anomalopia, and central scotoma, differ from those of cCSC, which is characterized by recurrent detachment, leading to local atrophy of the retinal pigment epithelium (RPE) and photoreceptors, potentially resulting in decreased vision or even blindness.^1^^,^^5^

Despite decades of research, the pathophysiology of CSC remains largely unknown. Indocyanine green angiography (ICGA) imaging has shown that focal choroidal capillaries delay filling and that surrounding dilated vessels cause hyperfluorescence.^6^^,^^7^ Advanced ultra-widefield (UWF) ICGA equipment has revealed that the dilated choroidal vessels in CSC eyes extend from the affected area to the vortex vein ampullae of the drainage system.^8^^,^^9^ Studies have also suggested that the asymmetric choroidal venous drainage with intervortex venous anastomoses may play a role in the development of CSC.^10^^,^^11^

The choroidal vascular bed has watershed zones situated between the vortex veins.^12^ The choroidal venous drainage system consists of four major vortex vein outflows divided along the vortex vein watershed zones (the boundary area between the territories of two distal adjacent veins).^13^ Each complete drainage system includes vortex veins, choroidal veins, and choroidal stroma. Compared to ICGA, swept-source optical coherence tomography angiography (OCTA) allows for volumetric assessments of the choroidal drainage system. The three-dimensional choroidal vascularity index (3D CVI), a general OCTA choroidal biomarker, is defined as the ratio of choroidal vascular volume (CVV) to choroidal volume.^14^ However, limited research has been conducted on the OCTA characterization of the vortex veins in eyes with CSC.

The purpose of this study was to explore how the choroidal vortex vein drainage system affects CSC utilizing UWF-OCTA. Accordingly, the choroidal vortex vein drainage system was analyzed in both individuals with CSC and healthy individuals. The proportion of the choroidal vortex vein drainage system (VV%) was measured along with the thickness of the choroid, the CVV, and the 3D CVI of the choroidal vortex vein drainage system (VV) in all four quadrants. Additionally, the study investigated the location correlation between the leakage points in fluorescein angiography (FA) and the vortex vein systems.

## Methods

### Study Design

This cross-sectional observational study was conducted from December 2021 to August 2022. The study enrolled 40 patients diagnosed with CSC from the Zhongshan Ophthalmic Center (ZOC) and 28 healthy volunteers as a control group. The study was approved by the medical ethics committee of ZOC, Sun Yat-sen University, China. All participants provided written informed consent, and all procedures followed the Declaration of Helsinki.

Eyes were diagnosed as CSC based on multimodal imaging findings: bullous serous detachments in fundus photography, SRD/pigment epithelium detachment in optical coherence tomography (OCT), RPE leakage as a “smokestack” or an “inkblot” pattern in FA, and dilated choroidal vessels in early-state ICGA with hyperpermeability in the intermediate phases.^5^ The inclusion criteria were as follows: (1) diagnosis as CSC according to fundus photography, FA, ICGA, OCT, and OCTA in the CSC group (individuals without ocular diseases in the control group); (2) 20 years ≤ age ≤ 60 years; (3) 22 mm ≤ axis length (AL) ≤ 26.5 mm; (4) –6.00 diopters (D) ≤ spherical equivalent ≤ 3.00 D; (5) 10 mm Hg ≤ intraocular pressure ≤ 21 mm Hg; and (6) OCT/OCTA image quality score (0–10) ≥7 and distinct leakage points in FA if CSC eyes had. The exclusion criteria were as follows: (1) a history of intraocular surgery or photodynamic therapy; (2) ocular diseases excluding CSC, such as macular edema, choroidal neovascularization, polypoid choroidal vasculopathy, uveitis, and glaucoma; (3) diabetic, hypertensive, and other systemic diseases affecting the choroidal circulation; (4) pregnancy and lactating; and (5) medication: allergy to sodium fluorescein or long-term use of corticosteroids or vasoactive medications. For patients with bilateral CSC, the lower-vision eyes were included, and the right eyes of healthy volunteers were included in the study.

### Data Collection

Basic patient information was collected, including gender, age, height, weight, blood pressure, medical history, and personal history. Comprehensive ophthalmologic examinations were performed, including best-corrected visual acuity measurement, intraocular pressure measurement (Topcon CT-1; Topcon, Tokyo, Japan), axis length measurement (IOLMaster 700; Carl Zeiss Meditec AG, Oberkochen, Germany), slit-lamp examination, dilated fundus photography (Canon CR-2; Canon, Tokyo, Japan), spectral-domain OCT (Heidelberg Spectralis, Heidelberg, Germany), and UWF-OCTA (TowardPi Medical Technology Co., Ltd, Beijing, China). FA (Heidelberg Spectralis), fundus autofluorescence photography, and ICGA (Heidelberg Spectralis) were also performed in the CSC group. All tests were completed on the same day (08:00–12:00, 14:00–17:00).

### Imaging Acquisition

Images of the choroidal layer were obtained using a UWF swept-source OCTA device (BM-400K BMizar; TowardPi Medical Technology Co., Ltd). The device is equipped with a swept-source vertical-cavity surface-emitting laser (VCSEL) laser with a wavelength of 1060 nm and has a scanning rate of up to 400,000 A-scans per second, providing lateral resolution of 10 µm and a depth resolution of 3.8 µm. The device is capable of 3D reconstruction and data analysis of the choroid. A 6 × 6-mm^2^ volumetric scan centered on the fovea was performed for the submacular choroid, and five 24 × 20-mm^2^ OCTA scans were obtained for the posterior fundus in five predefined locations: the central, superotemporal (ST), inferotemporal (IT), inferonasal (IN), and superonasal (SN) quadrants.^15^

To correct for any magnification errors caused by the AL, the actual size of the UWF-OCTA image was calculated using a previously verified formula (T = (AL-4) × S/20, where T is the actual size and S is the measured size).^16^ Manual adjustment of the segmentation was conducted after the automatic segmentation to obtain the most authentic choroid images. Two masked, trained researchers (KX and BW) participated in the image analysis to ensure the reliability and reproducibility of the study. If the two researchers could not reach a consensus, a third professor (SY) made the final evaluation. Additionally, three healthy eyes and three CSC eyes were examined and measured twice using the UWF-OCTA to assess reproducibility. The intraclass correlation coefficient (ICC) was calculated to evaluate the reproducibility of the OCTA data.

### The Proportion of the Choroidal Vortex Vein Drainage System (VV%)

**Figure 1.**
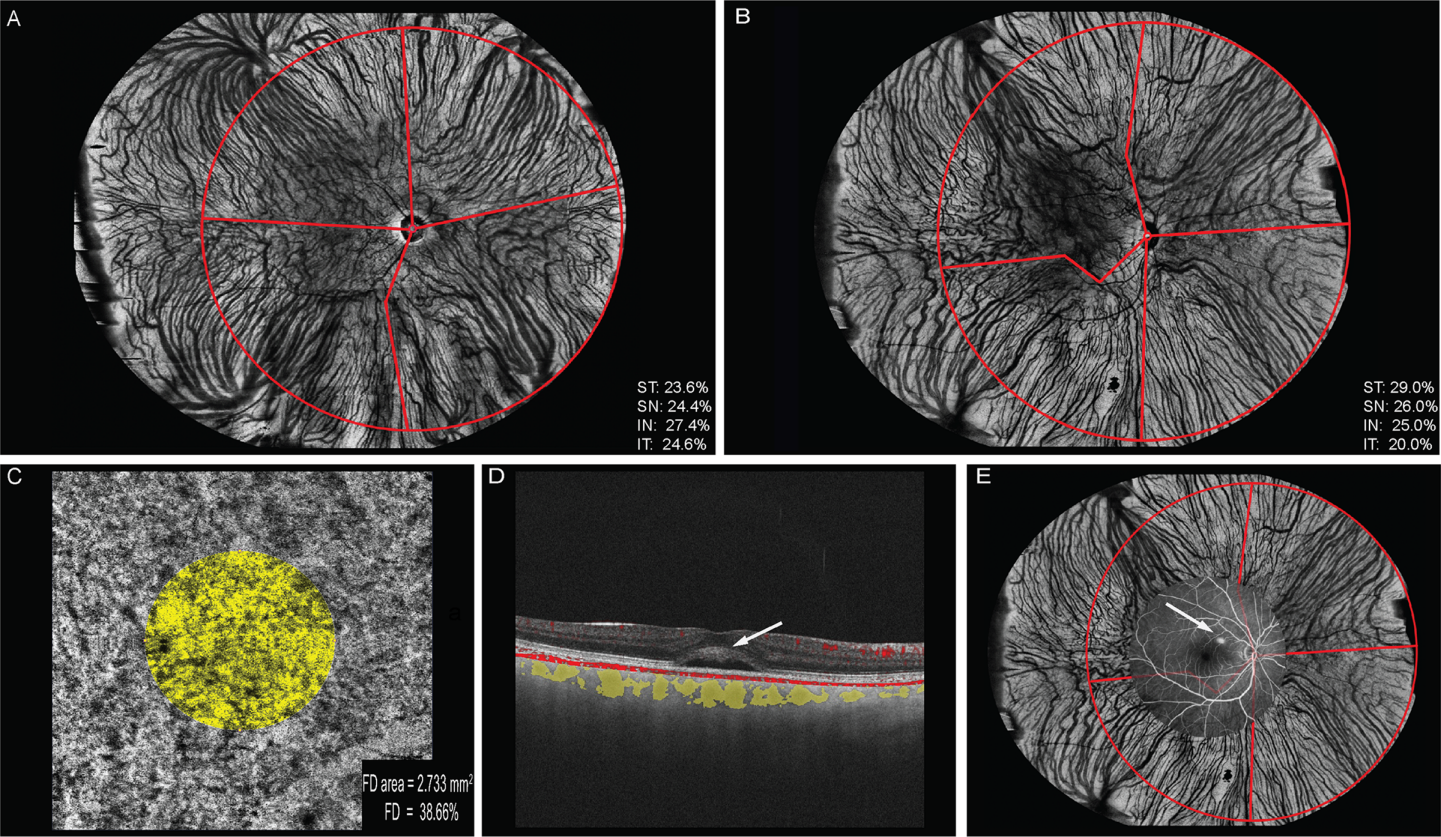
Imaging method. (**A**) Image of a 50-year-old healthy individual's right eye and (**B–E**) images of the right eye of a 43-year-old man with CSC. UWF-OCTA images of the choroid layer in the healthy eye (**A**) and CSC eye (**B**). In the CSC eye (**B**), choroidal vessels were dilated in the preferential drainage system (ST: VV% = 29.0%). (**C**) Centered on the fovea, the 6 × 6-mm^2^ swept-source OCTA choriocapillaris flow density image and the circular area (*yellow circle*, radius = 1.5 mm, FD = 38.66%) were obtained using the built-in software. (**D**) B-scan blood flow signal (*red*), serous retinal detachment (*white arrow*), and vessels (*yellow*) in Sattler's layer and Haller's layer of the 6-mm swept-source OCT horizontal scan centered on the fovea. (**E**) The FA image (55 degrees, venous phase) was colocated with the UWF choroid image. There was a leakage point (**E**, *white arrow*) colocated in the ST quadrant.

Five 24 × 20-mm^2^ en face OCT images were intelligently combined into a single UWF choroid image (>200 degrees), using the built-in montage function. ImageJ software V.1.53c (National Institutes of Health, Bethesda, MD, USA) was used to divide the entire postequatorial fundus of the UWF choroid image into four drainage quadrants: ST, SN, IN, and IT. This method was consistent with previous studies.^11^^,^^17^ The boundary of the whole postequatorial fundus was a circular area centered on the optic disc that intersected the posterior margin of the ST largest vortex vein. The fundus was divided into four drainage areas by vortex vein watersheds, with the watershed zone being the border between the territories of distribution of the terminal of two vortex veins (Figs. 1A, 1B).^11^^,^^17^^,^^18^ The VV% was defined as the ratio of the drainage area in each quadrant to the area of the entire postequatorial fundus.

### Choroidal Layer in the VV and Submacular Area

**Figure 2.**
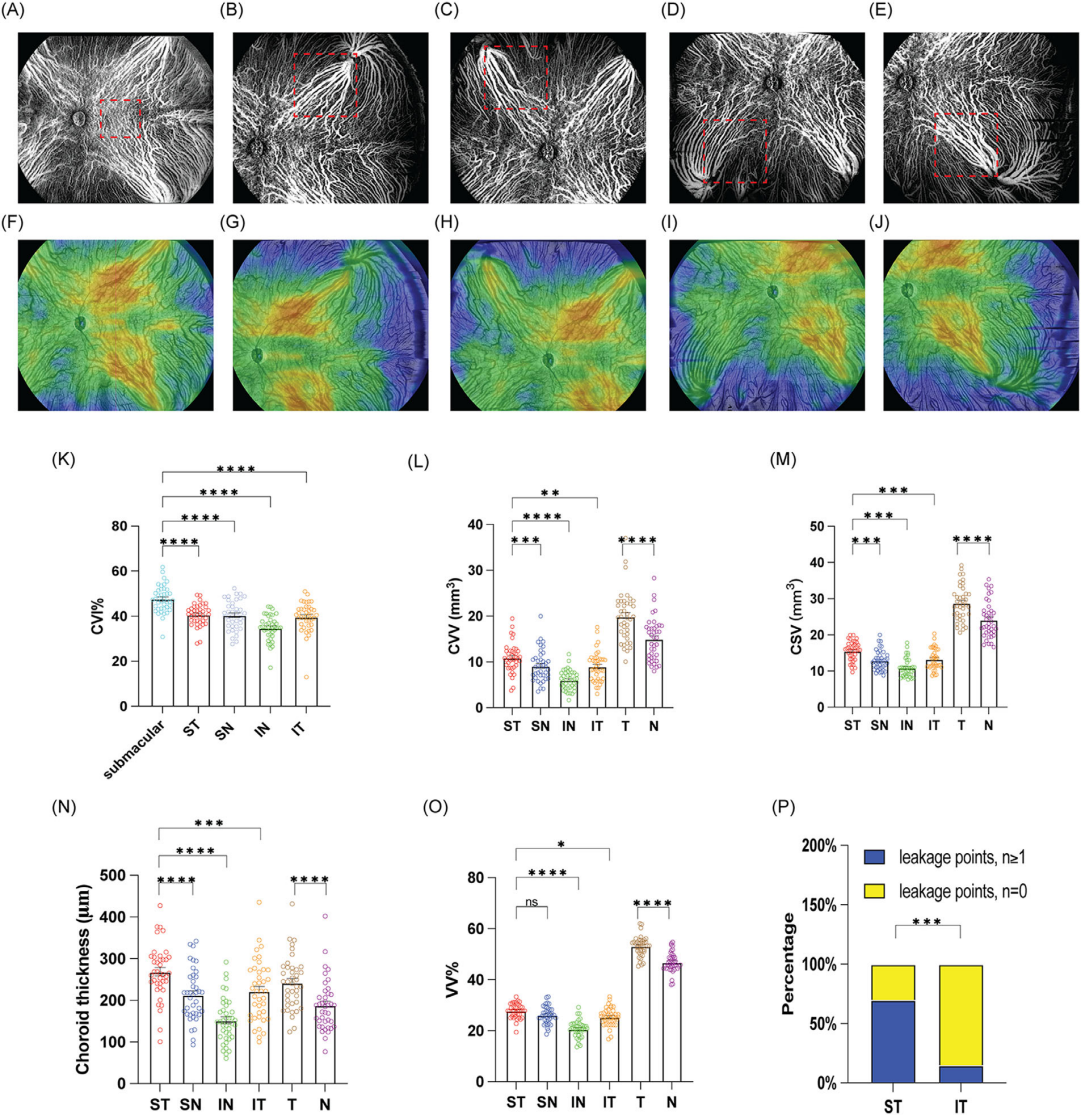
OCTA scan protocols. (**A–J**) Images of the left eye of a 50-year-old man with CSC in the central (**A**, **F**), ST (**B**, **G**), SN (**C**, **H**), IN (**D**, **I**), and IT (**E**, **J**) drainage quadrants, respectively. (**A–E**) Five different 24 × 20-mm^2^ OCTA choroid medium to large vessel images. (**A**) The 6 × 6-mm^2^ swept-source OCT volume scans (*red dotted box*) centered on the fovea. (**B–E**) The 9 × 9-mm^2^ swept-source OCT volume scans (*red dotted box* selected the center of the largest vortex vein ampulla as the vertice) in ST, SN, IN, and IT drainage quadrants, respectively. (**F–J**) The 24 × 20-mm^2^ choroidal thickness maps in the central, ST, SN, IN, and IT drainage quadrants, respectively. (**K**) Quantification of the CVI% of CSC eyes in the submacular, ST, SN, IN, and IT drainage quadrants is shown in the bar graphs. (**L–O**) Quantification of the choroidal parameters (CVV, CSV, choroid thickness, and VV%) of CSC eyes in ST, SN, IN, IT, temporal (T), and nasal (N) drainage quadrants is shown in the bar graphs. The data are reported as mean ± SEM, based on a repeated-measures one-way analysis of variance. (**P**) The leakage rate in the temporal quadrant is shown in the *bar graphs*. The data are percentages, based on a chi-square test. *P < 0.05, **P < 0.01, ***P < 0.001, and ****P < 0.0001. NS, not significant.

Given the choroidal structure, the choroidal layer (vessels and the stroma) in the VV and the submacular area were further analyzed. The border of the choroid was defined as the layer between the RPE–Bruch membrane complex and the choroid–sclera junction.^19^ The dark and light areas of the choroidal layer were defined as choroidal vessels and stroma, respectively, in the OCT images.^20^ In each quadrant, a 3D volumetric scan was performed, covering a 9 × 9-mm^2^ area, with the center of the largest vortex vein ampulla as the vertex and the diagonal pointing toward the posterior pole.^18^ The area of the vortex vein ampulla was measured using the device's built-in sketching tool (Supplementary Fig. S1a).^18^ The built-in software was used to obtain the choroidal thickness, choroidal volume (total light and dark area volume), choroidal vascular volume (total dark area volume), choroidal stromal volume (total light area volume), and 3D CVI (choroidal vascular volume/choroidal volume) (Figs. 2A–J).^20^ The CVV and CVI reflect the degree of choroidal vasodilation.

The choriocapillaris (CC) was defined as the layer between the RPE–Bruch membrane complex and 29 µm below the Bruch membrane.^21^ The CC flow density (FD) was automatically calculated by the built-in software and defined as the ratio of the light area to the circular area (radius = 1.5 mm) centered on the fovea (Fig. 1C).^21^ The projection of retinal vessels was removed by the device software. Swept-source OCT volume scans (6 × 6 mm^2^) were used to obtain the choroidal vascular volume and choroidal stromal volume centered on the fovea (Fig. 2A).

### Colocalization of the Leakage Points in FA and the Drainage System in En Face OCT

Adobe Photoshop CS6 (Adobe Systems, Inc., San Jose, CA, USA) was used to adjust the opacity of the FA (55 degrees, venous phase) images to 60%. To better understand the spatial relationship between the leakage points in FA and the relevant drainage quadrant, the FA image was registered with the UWF choroid image (Fig. 1E).

### Statistics

**Figure 3.**
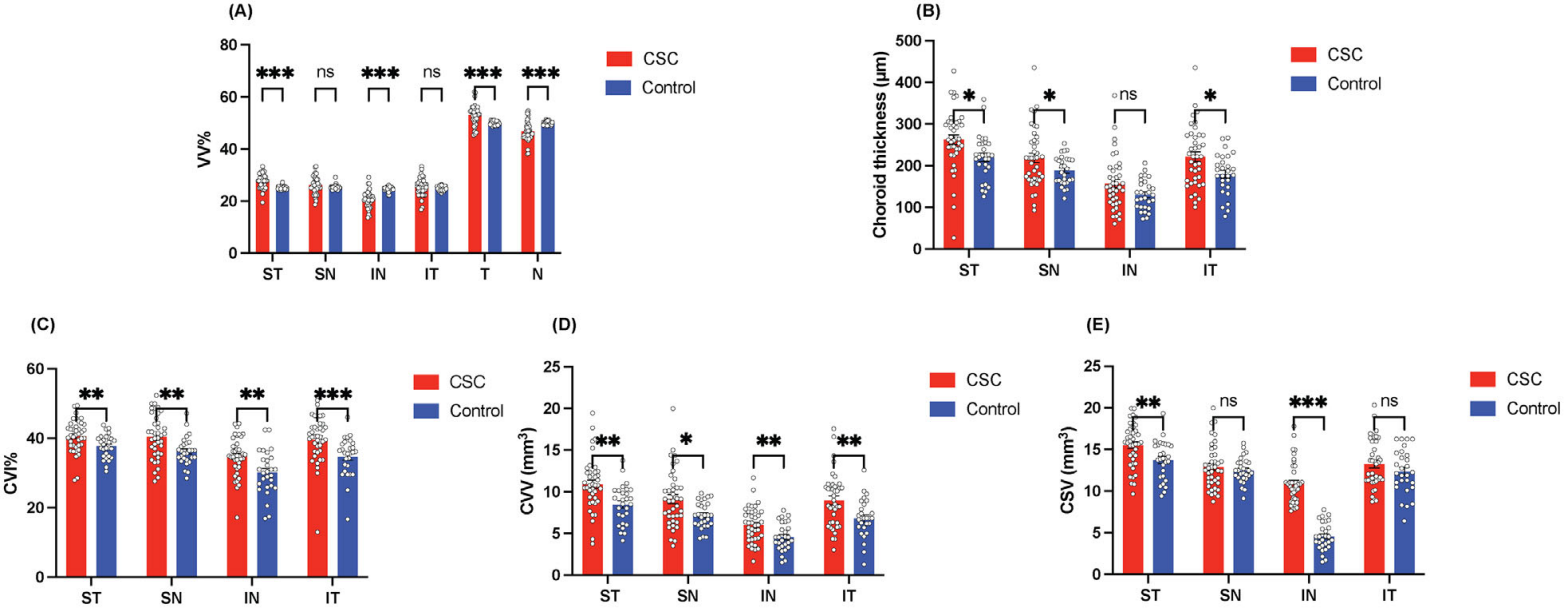
Comparison analysis between the CSC group and control group. (**A–E**) Quantification of the choroidal parameters (VV%, choroidal thickness, CVI, CVV, and CSV, respectively) is shown in the *bar graphs*. The data are reported as mean ± SEM, based on Mann–Whitney *U* tests. *P < 0.05, **P < 0.01, ***P < 0.001, and ****P < 0.0001.

The statistical analysis was conducted using Statistical Program for Social Sciences 26.0 (IBM SPSS Inc., New York, NY, USA). The normality of all continuous data was assessed using the Shapiro–Wilk test, and the equality of variances was confirmed with the Levene test. Quantitative values are reported as the mean ± SD (Tables 1^2^–3) or mean ± SEM (Figs. 2K–O and Figs. 3A–E). A *P* value of less than 0.05 was considered statistically significant. The comparison between patients with CSC and unaffected individuals was performed using independent sample *t* tests, Mann–Whitney *U* tests, and Pearson χ^2^ tests. The difference within groups was analyzed using a repeated-measures analysis of variance and paired *t* tests. The relationship between VV% and choroid vessel parameters (CVV and CVI) in the same quadrant and the CVV correlation between the submacular area and each quadrant were measured using either the Spearman or Pearson correlation.

**Table 1.** Demographic and Clinical Characteristics of Central Serous Chorioretinopathy Group

Demographics	Values
Gender, male/female	33 (82.5)/7 (17.5)
Medical history	
Mean time of disease, wk	16.29 ± 15.41
aCSC (SRD <12 wk)/cCSC (SRD ≥12 wk), eyes	21 (52.5)/19 (47.5)
Previous treatments, eyes	
Laser photocoagulation	8 (20.0)
Intravitreal anti-VEGF	0 (0)
None	32 (80.0)
Personal history	
Current/former/never smokers	6 (15.0)/5 (12.5)/29 (72.5)
Current/former/never drinkers	13 (32.5)/1 (2.5)/26 (65.0)
Bedtime after 11 pm	24 (60.0)
Ocular history	
BCVA (logMAR)	0.20 ± 0.21
Pseudophakic status	2 (5.0)

Values are presented as number (%) or mean ± SD.

BCVA, best-corrected visual acuity; logMAR, logarithm of the minimum angle of resolution; VEGF, vascular endothelial growth factor.

**Table 2.** Characteristics of Central Serous Chorioretinopathy Group and Control Group

Characteristic	CSC Group	Control Group	*P* Value
Demographics			
Number	40	28	
Age, y	43.70 ± 7.03 (22–55)	43.68 ± 12.26 (30–53)	0.370^a^
Male gender, *n* (%)	33 (82.5)	22 (78.6)	0.685^b^
Blood pressure			
Systolic, mm Hg	127.40 ± 13.74	126.07 ± 19.47	0.822^a^
Diastolic, mm Hg	83.30 ± 9.13	84.13 ± 10.74	0.755^a^
Height, cm	168.78 ± 6.20	169.19 ± 6.09	0.950^a^
Weight, kg	70.93 ± 21.50	66.63 ± 12.97	0.765^a^
Body mass index	24.83 ± 6.20	23.16 ± 3.68	0.533^a^
Ocular basic parameter			
Axial length, mm	23.85 ± 1.13	24.00 ± 1.09	0.950^a^
Intraocular pressure, mm Hg	13.41 ± 2.13	13.68 ± 2.15	0.562^a^

Values are presented as number (%), mean ± SD, or mean ± SD (range).

a *P* value determined by independent samples *t* test.

b *P* value determined by χ^2^ test.

**Table 3.** Choroidal Parameters of Central Serous Chorioretinopathy Group and Control Group

Characteristic	CSC Group	Control Group	*P* Value
VV%			
Superotemporal	27.78 ± 2.76	25.15 ± 0.72	**<0.001** **^a^**
Superonasal	26.12 ± 3.32	25.31 ± 0.93	0.126^a^
Inferonasal	20.71 ± 3.22	24.65 ± 0.91	**<0.001** **^a^**
Inferotemporal	25.40 ± 3.46	24.87 ± 0.84	0.315^a^
*P* value	**<0.001** **^b^**	0.270^b^	
Temporal	53.17 ± 3.82	50.02 ± 0.75	**<0.001** **^a^**
Nasal	46.83 ± 3.82	49.97 ± 0.75	**<0.001** **^a^**
*P* value	**<0.001** **^c^**	0.862^c^	
Choroidal thickness, µm			
Superotemporal	268.68 ± 64.79	220.33 ± 55.60	**0.014** **^a^**
Superonasal	212.92 ± 61.76	188.33 ± 34.37	**0.040** **^a^**
Inferonasal	151.96 ± 53.82	129.96 ± 36.37	0.108^a^
Inferotemporal	221.86 ± 72.67	179.74 ± 49.28	**0.021** **^a^**
*P* value	**<0.001** **^b^**	**<0.001** **^b^**	
CVI, %			
Superotemporal	40.60 ± 4.66	37.68 ± 3.40	**0.006** **^a^**
Superonasal	40.43 ± 6.31	36.23 ± 4.01	**0.003** **^a^**
Inferonasal	35.17 ± 6.31	30.07 ± 6.30	**0.001** **^a^**
Inferotemporal	39.68 ± 6.55	33.56 ± 7.79	**<0.001** **^a^**
*P* value	**<0.001** **^b^**	**<0.001** **^b^**	
CVV, mm^3^			
Superotemporal	10.91 ± 3.19	8.49 ± 2.33	**0.001** **^a^**
Superonasal	9.12 ± 3.43	7.19 ± 1.57	**0.017** **^a^**
Inferonasal	6.00 ± 0.33	4.55 ± 1.68	**0.005** **^a^**
Inferotemporal	8.98 ± 3.22	6.83 ± 2.31	**0.005** **^a^**
*P* value	**<0.001** **^b^**	**<0.001** **^b^**	
CSV, mm^3^			
Superotemporal	15.52 ± 2.62	13.73 ± 2.35	**0.005** **^a^**
Superonasal	12.91 ± 2.68	12.49 ± 1.52	0.827^a^
Inferonasal	10.89 ± 0.83	10.14 ± 1.56	**<0.001** **^a^**
Inferotemporal	13.25 ± 2.89	12.36 ± 2.78	0.204^a^
*P* value	**<0.001** **^b^**	**<0.001** **^b^**	
Submacular area			
CVV, mm^3^	6.97 ± 3.26	5.33 ± 2.10	**0.022** **^a^**
CSV, mm^3^	7.24 ± 2.12	6.30 ± 1.55	**0.049** **^a^**
CVI, %	47.63 ± 5.93	44.29 ± 7.13	**0.047** **^a^**
CC FD, %	36.81 ± 6.17	39.18 ± 4.00	**0.030** **^a^**

Values are presented as mean ± SD. Significant *P* values are displayed in bold.

a *P* value determined by Mann–Whitney *U* test.

b *P* value determined by repeated-measures analysis of variance.

c *P* value determined by paired *t* test.

## Results

### Population Characteristics

Eighty-three participants were initially screened for the study. However, 15 individuals were excluded due to unknown medical history (two cases), poor quality of OCTA imaging (five cases), and incomplete data (eight cases). After the stringent screening, the study included 40 eyes of 40 patients with CSC and 28 eyes of 28 healthy volunteers. No significant differences were observed in baseline characteristics between the CSC and control groups. The mean age of the CSC group was 43.70 ± 7.03 years (range, 22–55), with 33 (82.5%) males and 7 (17.5%) females. Of the patients with CSC, 60% had a history of staying up late before onset, 27.5% had a history of smoking, and 35% had a history of drinking. Demographic and clinical details of the CSC group are presented in Tables 1 and 2. Further, the reliability and repeatability of all OCT and OCTA data were assessed and expressed as ICCs (Supplementary Table S1).

### The Proportion of Choroidal Vortex Vein Drainage System (VV%)

The comparison of VV% between the CSC group and the control group is summarized in Table 3. As expected, in the CSC group, statistically significant differences were noted in the VV% among the drainage quadrants (ST, 27.78% ± 2.76%; SN, 26.12% ± 3.32%; IN, 20.71% ± 3.22%; IT, 25.40% ± 3.46%; *P* < 0.001). In contrast, the control group showed no significant differences in the VV% of each drainage quadrant (ST, 25.15% ± 0.72%; SN, 25.31% ± 0.93%; IN, 24.65% ± 0.91%; IT, 24.87% ± 0.84%; *P* = 0.270). The results showed that the CSC group had a higher proportion of the choroidal vortex vein drainage system in the ST quadrant compared to the control group (*P* < 0.001). Meanwhile, the proportion of the drainage area in IN in the CSC group was significantly lower compared to the control group (*P* < 0.001). A paired *t* test revealed that the temporal VV% was significantly higher than the nasal VV% (*P* < 0.001) in the CSC group but not in the control group (*P* = 0.862).

### Choroidal Layer in Each VV Quadrant

Table 3 presents a comparison of choroidal thickness between the CSC and control groups. In the ST, SN, and IT quadrants, the choroidal layer was significantly thicker in the CSC eyes than in the normal eyes (all *P* < 0.05). The within-group analysis of choroidal thickness showed statistically significant differences in both the CSC group (*P* < 0.001) and the control group (*P* < 0.001). In eyes with CSC, the thicker choroidal layer occurred in the temporal vortex vein systems (Fig. 2N; temporal, 242.30 ± 63.19 µm vs. nasal, 187.90 ± 61.73 µm; *P* < 0.0001). The CVI data (Table 3) showed that the CVI was significantly higher in the eyes of patients with CSC in each quadrant compared to the healthy eyes (all *P* < 0.05). Similarly, the CSC group had significantly larger choroidal vascular volume in all regions compared to the healthy eyes (Table 3, all *P* < 0.05). Further, in CSC eyes, both CVV and CSV in the temporal drainage quadrant were higher than that in the nasal drainage quadrant (Figs. 2L–M, all *P* < 0.0001). Interestingly, for CSC eyes, the ST drainage quadrant had the largest CVV and CSV as well as the thickest choroidal layer among the four drainage quadrants (Figs. 2L–N, all *P* < 0.05), indicating that this quadrant contributes to the major choroid drainage in CSC.

### Choroidal Layer in the Submacular Area

In the submacular area, the CSC group had greater choroidal vascular volume, greater choroidal stromal volume, and higher CVI than the control group (all *P* < 0.05), as shown in Table 3. However, the CC flow density in the submacular area of the CSC group was lower compared to that in the control group (*P* = 0.03). Additionally, the submacular region also had the highest CVI (Fig. 2K, all *P* < 0.05).

### The Number of Leakage Points and the Leakage Rate in the Temporal Quadrant

The number of leakage points and the leakage rates in the temporal quadrant are shown in Figure 2P. There were 37 leakage points in the ST quadrant and 6 in the IT quadrant. Compared with the IT drainage quadrant, the ST drainage quadrant had a significantly higher leakage rate (Fig. 2P, 70% vs. 15%; *P* < 0.001).

### Correlation Analysis

**Figure 4.**
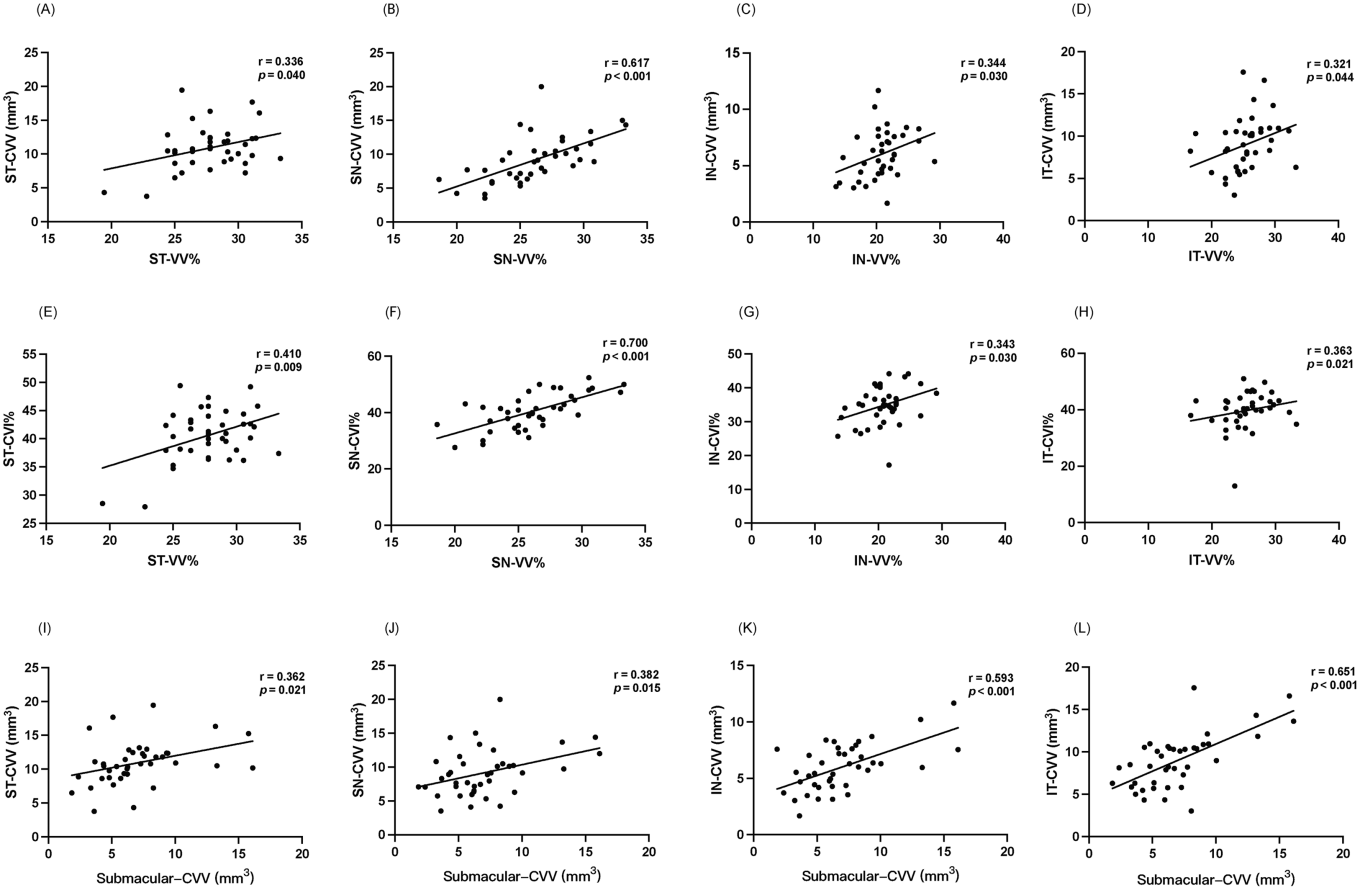
Correlation analysis. (**A–H**) Univariate correlation analysis between VV% and choroid vessel parameters (CVV and CVI) in each quadrant (all *r* > 0.300, *P* < 0.05). (**I–L**) Univariate correlation analysis in CVV between the submacular area and each quadrant (all *r* > 0.350, *P* < 0.05).

Notably, a positive correlation was observed between CVI and the ratio of the drainage area in each quadrant (Figs. 4E–H; ST, *r* = 0.410; SN, *r* = 0.700; IN, *r* = 0.343; IT, *r* = 0.363; all *P* < 0.05). Similarly, there was a significant positive correlation between CVV and the ratio of the drainage area in each quadrant (ST, *r* = 0.336; SN, *r* = 0.617; IN, *r* = 0.344; IT, *r* = 0.321; all *P* < 0.05), as shown in Figures 4A–D. Additionally, the dilated vessels in the submacular area were positively associated with dilated vortex veins in all quadrants (Figs. 4I–L, all *r* > 0.350, *P* < 0.05).

## Discussion

In this study, we evaluated the choroidal vortex vein drainage system in two and three dimensions using advanced UWF-OCTA technology. Our results demonstrated that the choroidal vortex vein drainage system in eyes with CSC was significantly more asymmetric compared to healthy eyes, which is in line with previous findings indicating that asymmetric choroidal venous outflow is a common characteristic of CSC eyes.^10^^,^^17^

Based on our results, the proportion of the temporal drainage area was 53.17% ± 3.82%, which was significantly higher than the nasal drainage area (46.83% ± 3.82%), and the smallest drainage area was the IN (20.71% ± 3.22%). These findings are consistent with previous studies by Bacci et al.^11^ and Ramtohul et al.,^17^ who found similar patterns of asymmetric choroidal vortex vein drainage in CSC eyes. Additionally, Jung et al.^10^ found that the brightness level of the IT quadrant was highest among all regions when quantifying UWF ICGA brightness levels of CSC eyes using Otsu's method. These findings suggest that the dominant choroidal drainage area in CSC eyes is likely in the temporal quadrant. Based on in vivo studies, Hayreh^22^^,^^23^ demonstrated that the vortex veins have a segmental distribution in the choroid and are divided into four functionally independent areas by watershed zones. Verma et al.^24^ found no significant relationship between the distribution or location of vortex vein ampullae and various demographic factors, including age, gender, and ethnicity. Mori and coworkers^25^ also presented evidence of asymmetrical choroidal venous drainage in some healthy individuals. Thus, it can be hypothesized that CSC eyes may have developmental anomalies in the asymmetric spatial arrangement of venous drainage routes but not necessarily pathogenic ones.

Further, the choroidal thickness of vortex vein ampullas and the intrascleral portion of vortex veins were analyzed. Regarding choroidal thickness, there was a significant difference between the CSC group and the control group in the ST, SN, and IT quadrants, with the majority of the choroidal thickening located in the temporal drainage area. This finding is also in agreement with those of Bacci et al.^11^ We must explain the interesting finding that in temporal quadrant, CSC eyes have bigger drainage areas and thicker choroidal layers. The choroid is a vascularized membrane structure, and the asymmetric choroidal thickening may be attributed to the imbalanced distribution of choroidal venous flow.

We further analyzed the choroidal vessels and found that CSC eyes had a greater CVI and CVV in all drainage quadrants, indicating dilation of the choroidal vortex veins along the entire drainage system, not just in the submacular area. Some research has shown that inflammation, mineralocorticoid receptor activation, hemodynamic changes, and genetic predispositions may play a role in regulating choroidal blood flow.^26^ High levels of corticosteroids (i.e., mineralocorticoids and glucocorticoids) have been identified as an important risk factor for the development and recurrence of CSC.^27^^,^^28^ The mineralocorticoid receptor is expressed in most choroidal endothelial cells and induces vessel dilation and leakage.^29^^,^^30^ However, these factors cannot fully explain the asymmetric choroidal thickening because all choroidal vessels may be affected. Interestingly, we also found that the greatest choroid blood flow occurred in the temporal quadrants, especially the ST drainage area, and the choroidal blood flow was proportionate to the drainage area (Figs. 4A–H). Thus, the asymmetric choroidal vortex vein drainage system may play a key role in the imbalanced distribution of choroidal venous flow. The choroid blood outflowed along the choroidal vortex vein drainage system in each quadrant and out to the ampullae. The dominant ST system may drain more blood flow out to the sclera and lead to the imbalanced distribution of choroidal venous flow and venous overload. In sum, we think that some factors first lead to the hyperperfusion of the whole choroid, and the anatomically imbalanced drainage system then results in the imbalanced distribution of venous flow.

However, it remains unclear why SRD primarily occurs in the macular area in CSC. Interestingly, our results indicated a positive correlation between the choroidal vessels in the submacular area and the dilation of vortex veins, suggesting that the vortex vein's drainage function in all four quadrants affects the submacular choroidal blood perfusion (Figs. 4I–L). The macular area is located in the temporal drainage quadrant, and the leakage rate was significantly higher in the ST quadrant than in the IT quadrant. Moreover, most of the choroid blood flow was concentrated in the temporal quadrants, especially the ST drainage area, suggesting that the ST choroidal vortex vein drainage system may primarily control the venous outflow in the submacular choroid of CSC eyes. Recent studies have shown that intervortex venous anastomoses mainly involve the temporal vortex vein systems near the macular area in CSC eyes, and venous overload in the submacular area may be redistributed by these anastomoses to restore balance.^31^^,^^32^ The lower CC FD found in patients with CSC suggests choriocapillaris hypoperfusion. Thus, the dominant ST drainage system leads to chronic venous congestion in the submacular region, dilation of large- and medium-sized choroidal vessels, compression of the choriocapillaris and ischemia, dysfunction of the RPE, and subsequent RPE leakage.^33^^–^^35^

Our study had several limitations, including the small sample size and the large age interval (20–60 years). Further, the evaluated leakage points were confined to the macular area. The cross-sectional design did not allow for an accurate analysis of causality between the choroidal vortex vein drainage system and CSC, and limitations in the current technology affected assessment of the fundus beyond the postequator area. Moreover, some CSC eyes received laser photocoagulation treatment, which could influence the choroidal vortex vein drainage system.

In summary, our research indicates that eyes with CSC may have an unevenly distributed drainage system for the choroidal vortex vein. The entire drainage system appears to have excessive blood flow, with a greater concentration in the dominant drainage quadrant (ST), which includes the submacular area. Thus, the preferred route for draining the submacular choroid in CSC eyes may be through the ST drainage system. To confirm these findings, further studies are needed to provide more evidence regarding the developmental irregularities in the choroidal vortex vein drainage system in CSC.

## Summary

In eyes with CSC, choroidal thickening is related to the choroidal venous dilation and a delay in vascular filling of the choroid. UWF-OCTA is a reliable noninvasive tool for analyzing the choroidal layer.

In CSC eyes, the asymmetric vortex vein drainage system may contribute to choroidal venous overload. Serous retinal detachment primarily occurs in the macular area because the ST drainage system is the preferential drainage route of the submacular choroid.

## Supplementary Material

Supplement 1

Supplement 2

## References

[1] Liew G, Quin G, Gillies M, Fraser-Bell S. Central serous chorioretinopathy: a review of epidemiology and pathophysiology. *Clin Exp Ophthalmol*. 2013; 41: 201–214. 22788735 10.1111/j.1442-9071.2012.02848.x

[2] Kitzmann AS, Pulido JS, Diehl NN, Hodge DO, Burke JP. The incidence of central serous chorioretinopathy in olmsted county, minnesota, 1980-2002. *Ophthalmology*. 2008; 115: 169–173. 18166410 10.1016/j.ophtha.2007.02.032

[3] Chhablani J, Cohen FB. Central serous chorioretinopathy international group. multimodal imaging-based central serous chorioretinopathy classification. *Ophthalmol Retina.* 2020; 4: 1043–1046. 33131671 10.1016/j.oret.2020.07.026

[4] Gemenetzi M, De Salvo G, Lotery AJ. Central serous chorioretinopathy: an update on pathogenesis and treatment. *Eye (Lond)*. 2010; 24: 1743–1756. 20930852 10.1038/eye.2010.130

[5] Kaye R, Chandra S, Sheth J, et al. Central serous chorioretinopathy: an update on risk factors, pathophysiology and imaging modalities. *Prog Retin Eye Res*. 2020; 79: 100865. 32407978 10.1016/j.preteyeres.2020.100865

[6] Hayashi K, Hasegawa Y, Tokoro T. Indocyanine green angiography of central serous chorioretinopathy. *Int Ophthalmol*. 1986; 9: 37–41. 3721709 10.1007/BF00225936

[7] Quin G, Liew G, Ho IV, Gillies M, Fraser-Bell S. Diagnosis and interventions for central serous chorioretinopathy: review and update. *Clin Exp Ophthalmol*. 2013; 41: 187–200. 22788713 10.1111/j.1442-9071.2012.02847.x

[8] Pang CE, Shah VP, Sarraf D, Freund KB. Ultra-widefield imaging with autofluorescence and indocyanine green angiography in central serous chorioretinopathy. *Am J Ophthalmol*. 2014; 158: 362–371. 24794091 10.1016/j.ajo.2014.04.021

[9] Hirahara S, Yasukawa T, Kominami A, Nozaki M, Ogura Y. Densitometry of choroidal vessels in eyes with and without central serous chorioretinopathy by wide-field indocyanine green angiography. *Am J Ophthalmol*. 2016; 166: 103–111. 27059632 10.1016/j.ajo.2016.03.040

[10] Jung JJ, Yu DJG, Ito K, et al. Quantitative assessment of asymmetric choroidal outflow in pachychoroid eyes on ultra-widefield indocyanine green angiography. *Invest Ophthalmol Vis Sci*. 2020; 61: 50. 10.1167/iovs.61.8.50 PMC7425745 32735325

[11] Bacci T, Oh DJ, Singer M, Sadda S, Freund KB. Ultra-widefield indocyanine green angiography reveals patterns of choroidal venous insufficiency influencing pachychoroid disease. *Invest Ophthalmol Vis Sci*. 2022; 63: 17. 10.1167/iovs.63.1.17 PMC8762674 35019945

[12] Hayreh SS. In vivo choroidal circulation and its watershed zones. *Eye (Lond)*. 1990; 4(pt 2): 273–289. 2199236 10.1038/eye.1990.39

[13] Hayreh SS. Posterior ciliary artery circulation in health and disease: the Weisenfeld lecture. *Invest Ophthalmol Vis Sci*. 2004; 45: 749–748. 14985286 10.1167/iovs.03-0469

[14] Sun G. New insights into the association between choroidal vessels and choriocapillaris in normal eyes. *Retina*. 2021; 41: 2612–2619. 34173364 10.1097/IAE.0000000000003238

[15] Wang Y, Lai Y, Zhou X, et al. Ultra-wide-field optical coherence tomography angiography in mild familial exudative vitreoretinopathy [published online January 31, 2023]. *Retina*. 10.1097/IAE.0000000000003754 36809312

[16] Zeng Q, Yao Y, Tu S, Zhao M. Quantitative analysis of choroidal vasculature in central serous chorioretinopathy using ultra-widefield swept-source optical coherence tomography angiography. *Sci Rep*. 2022; 12: 18427. 36319689 10.1038/s41598-022-23389-1 PMC9626581

[17] Ramtohul P, Cabral D, Oh D, Galhoz D, Freund KB. En face ultrawidefield OCT of the vortex vein system in central serous chorioretinopathy. *Ophthalmol Retina*. 2023; 7: 346–353. 36228952 10.1016/j.oret.2022.10.001

[18] Funatsu R, Terasaki H, Shiihara H, et al. Quantitative evaluations of vortex vein ampullae by adjusted 3D reverse projection model of ultra-widefield fundus images. *Sci Rep*. 2021; 11: 8916. 33903616 10.1038/s41598-021-88265-w PMC8076294

[19] Nickla DL, Wallman J. The multifunctional choroid. *Prog Retin Eye Res*. 2010; 29: 144–168. 20044062 10.1016/j.preteyeres.2009.12.002 PMC2913695

[20] Betzler BK, Ding J, Wei X, et al. Choroidal vascularity index: a step towards software as a medical device. *Br J Ophthalmol*. 2022; 106: 149–155. 33514528 10.1136/bjophthalmol-2021-318782

[21] Chu Z, Zhang Q, Gregori G, Rosenfeld PJ, Wang RK. Guidelines for imaging the choriocapillaris using OCT angiography. *Am J Ophthalmol*. 2021; 222: 92–101. 32891694 10.1016/j.ajo.2020.08.045 PMC7930158

[22] Hayreh SS. Segmental nature of the choroidal vasculature *Br J Ophthalmol* 1975; 59: 631–648. 812547 10.1136/bjo.59.11.631 PMC1017426

[23] Hayreh SS. Physiological anatomy of the choroidal vascular bed. *Int Ophthalmol*. 1983; 6: 85–93. 6832901 10.1007/BF00127636

[24] Verma A, Maram J, Alagorie AR, et al. Distribution and location of vortex vein ampullae in healthy human eyes as assessed by ultra-widefield indocyanine green angiography. *Ophthalmol Retina*. 2020; 4: 530–534. 31964607 10.1016/j.oret.2019.11.009

[25] Mori K, Gehlbach PL, Yoneya S, Shimizu K. Asymmetry of choroidal venous vascular patterns in the human eye. *Ophthalmology*. 2004; 111: 507–512. 15019327 10.1016/j.ophtha.2003.06.009

[26] Spaide RF, Gemmy Cheung CM, Matsumoto H, et al. Venous overload choroidopathy: a hypothetical framework for central serous chorioretinopathy and allied disorders. *Prog Retin Eye Res*. 2022; 86: 100973. 34029721 10.1016/j.preteyeres.2021.100973

[27] Zhao M, Celerier I, Bousquet E, et al. Mineralocorticoid receptor is involved in rat and human ocular chorioretinopathy. *J Clin Invest*. 2012; 122: 2672–2679. 22684104 10.1172/JCI61427 PMC3386817

[28] Yan W, Long P, Zhang L, et al. The temporal topography of central serous chorioretinopathy in the chinchilla rabbits induced by intravenous injection of adrenaline: an in vivo study. *Drug Des Devel Ther.* 2022; 16: 3275–3283. 10.2147/DDDT.S381957 PMC9514269 36177348

[29] Yu S, Cui K, Wu P, et al. Melatonin prevents experimental central serous chorioretinopathy in rats. *J Pineal Res*. 2022; 73: e12802. 35436360 10.1111/jpi.12802

[30] Behar-Cohen F, Zhao M. Corticosteroids and the retina: a role for the mineralocorticoid receptor. *Curr Opin Neurol*. 2016; 29: 49–54. 26679569 10.1097/WCO.0000000000000284

[31] Matsumoto H, Hoshino J, Mukai R, et al. Vortex vein anastomosis at the watershed in pachychoroid spectrum diseases. *Ophthalmol Retina*. 2020; 4: 938–945. 32651158 10.1016/j.oret.2020.03.024

[32] Brinks J, van Dijk EHC, Meijer OC, Schlingemann RO, Boon CJF. Choroidal arteriovenous anastomoses: a hypothesis for the pathogenesis of central serous chorioretinopathy and other pachychoroid disease spectrum abnormalities. *Acta Ophthalmol*. 2022; 100: 946–959. 35179828 10.1111/aos.15112 PMC9790326

[33] Yang L, Jonas JB, Wei W. Optical coherence tomography-assisted enhanced depth imaging of central serous chorioretinopathy. *Invest Ophthalmol Vis Sci*. 2013; 54: 4659–4665. 23737472 10.1167/iovs.12-10991

[34] Razavi S, Souied EH, Cavallero E, Weber M, Querques G. Assessment of choroidal topographic changes by swept source optical coherence tomography after photodynamic therapy for central serous chorioretinopathy. *Am J Ophthalmol*. 2014; 157: 852–860. 24412124 10.1016/j.ajo.2013.12.029

[35] Daruich A, Matet A, Dirani A, et al. Central serous chorioretinopathy: recent findings and new physiopathology hypothesis. *Prog Retin Eye Res*. 2015; 48: 82–118. 26026923 10.1016/j.preteyeres.2015.05.003

