# Supplementary material for: Choroidal Vortex Vein Drainage System in Central Serous Chorioretinopathy Using Ultra-Widefield Optical Coherence Tomography Angiography
Source: Transl Vis Sci Technol. 2023 Sep 22;12(9):17. doi: 10.1167/tvst.12.9.17 (PMC10519436; doi:10.1167/tvst.12.9.17)
Supplement: Supplement 1 [file tvst-12-9-17_s001.pdf]

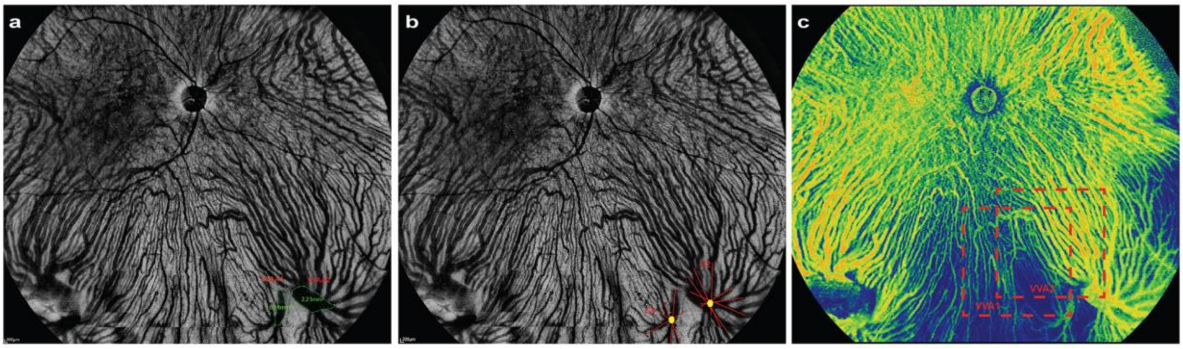

Figure S1. Step-by-step illustrations of confirming the largest vortex vein ampulla. (**a**) First, in each quadrant, the ends of each vortex vein branch were connected with a smooth curve and the area enclosed by the curve was defined as the area of the vortex vein ampulla (VVAA). The VVAA was measured by the built-in sketching tool. VVAA1 = 0.94 mm^2, VVAA2 = 2.23mm^2. (**b**) Second, the intersection or the center (yellow circle) formed by the straight line (red line) drawn from the thick blood vessel was defined as the location of the center of vortex vein ampulla (VVA). C1: the center of VVA1; C2: the center of VVA2. (**c**) Third, we further analyzed the CVV, CSV, and CVI of each vortex vein (a 3D volumetric scan was performed covering a 9 × 9 mm^2 area, with the center of each vortex vein ampulla as the vertex and the diagonal pointing toward the posterior pole) in order to make sure the largest vortex vein ampulla again. The bigger area vortex vein ampulla had higher choroidal parameters. VVA1: CVV = 3.807 mm^3; CSV = 8.667 mm^3; CVI = 30.52%. VVA2: CVV = 6.363 mm^3; CSV = 9.081 mm^3; CVI = 41.20%. VVA2 was the largest vortex vein ampulla in this quadrant. CVV, choroidal vascular volume; CSV, choroidal stromal volume; CVI, choroidal vascularity index.
